# Intrinsic Functional Connectivity Alterations of the Fusiform Face Area in Autism Spectrum Disorder

**DOI:** 10.3390/neurosci6020029

**Published:** 2025-04-01

**Authors:** Natalia Kleinhans, Sarah F. Larsen, Annette Estes, Elizabeth Aylward

**Affiliations:** 1 Department of Radiology, University of Washington, Seattle, WA 98109, USA; salarsen@uw.edu; 2 Integrated Brain Imaging Center, University of Washington, Seattle, WA 98195, USA; 3 Institute on Human Development and Disability, University of Washington, Seattle, WA 98195, USA; estesa@uw.edu; 4 Department of Speech and Hearing Sciences, University of Washington, Seattle, WA 98195, USA; 5 Seattle Children’s Research Institute, Seattle, WA 98109, USA

**Keywords:** overconnectivity, fusiform face area, resting state, autistic, face memory, face processing

## Abstract

Intrinsic connectivity of the fusiform face area (FFA) was assessed using resting-state functional magnetic resonance imaging (fMRI) to compare adults with autism spectrum disorder (ASD; *n* = 17) and age-, sex-, and IQ-matched typically developing controls (TD; *n* = 22). The FFA seed region was delineated in each participant using a functional localizer task. Whole brain analyses of FFA connectivity revealed increased connectivity between the right FFA and the vermis, sensorimotor cortex, and extended face-processing network in individuals with ASD compared to TD participants; the TD group did not demonstrate increased functional connectivity. No group differences were observed from the left FFA. The relationship between FFA connectivity and the ability to remember faces significantly differed between the groups. Better face memory performance was positively correlated with increased connectivity within general visual processing areas in the ASD participants; whereas for the TD group, better face memory performance was associated with increased connectivity with brain regions related to face encoding, recognition, and retrieval. FFA overconnectivity with face, emotion, and memory processing areas, along with atypical relationships between FFA–occipito-temporal connections and face memory performance highlights a possible mechanism underlying social dysfunction in individuals with ASD.

## 1. Introduction

Autism spectrum disorder (ASD) is a neurodevelopmental condition characterized by deficits in social communication skills and restricted, repetitive behaviors and interests. Social deficits in ASD may be mediated by abnormalities in face processing, which emerge during infancy [1,2]. Performance differences in face processing include impaired memory for faces, but not for objects [3]; a reduced impact of inversion when processing faces [4,5,6]; and altered eye movement patterns [7,8,9]. Impaired face processing can limit the acquisition and retention of contextual information during social interactions through mechanisms that subserve processing social stimuli, such as eye gaze, joint attention, emotion perception, and face recognition, impacting social interaction.

Neuroimaging studies of face perception in ASD have primarily used blood oxygenation level-dependent (BOLD) fMRI responses during task performance to probe regional neural activation during face processing. Initial studies focused on the fusiform face area (FFA), a region thought to play a specialized (though not exclusive) role in face processing [10], and suggested that abnormal development of the FFA was present in individuals with ASD. Functional imaging investigations of this region initially suggested a reduction or lack of FFA activation in ASD during face processing [11,12,13,14], whereas later studies found a more complex relationship that was influenced by the comparison population, specific demands of the task, and amount of time spent fixating on the eyes [12,14,15,16,17,18,19,20,21].

The opportunity to non-invasively measure the integrity of brain connectivity without task-related confounds has led to a surge in the application of resting-state fMRI approaches. In both healthy populations and a number of disease states, spontaneous patterns of neural activity present during the absence of a task have been shown to correspond with functional brain networks [22]. Previous studies have shown that the core face-processing network is functionally correlated during the resting state [23,24] and is associated with behavioral measures of face processing [25,26]. In ASD, in-depth research on the intrinsic connectivity of the major resting-state networks has been advanced by the Autism Brain Imaging Data Exchange [27]. Yet, to our knowledge, very few studies on intrinsic FFA connectivity in ASD have been reported. To address this gap, we conducted a resting-state functional connectivity study in young adults with ASD using individually defined FFAs as the seed regions. Given that the FFA is specifically involved in the perception of unique face identities within the core system for face processing, we hypothesized that individual differences in intrinsic connectivity from this region might be related to difficulties autistic individuals have with the integration of face perception and memory. Based on the decreased recruitment of brain regions within core and extended face networks observed in ASD during BOLD fMRI assessments of face perception [28], and previous resting-state studies that indicate underconnectivity between core regions within the network [29], we hypothesized that individuals with ASD would show reduced connectivity between the FFA and regions of the core and extended face-processing networks. To assess whether intrinsic connectivity patterns were related to atypical face processing, we measured face memory in our ASD and TD participants. Because memory for socially relevant information appears to be specifically impaired in ASD [3] and possibly related to underconnectivity, we further hypothesized that face memory performance would be positively correlated to resting connectivity between the FFA and regions involved in facial perception, encoding, and retrieval, such as the amygdala, superior temporal gyrus, orbital frontal gyrus, and cerebellum (socioemotional processing); the hippocampus, parahippocampal gyrus, posterior cingulate, and precuneus (encoding and retrieval); and occipitotemporal areas (visual perception) [30,31,32], indicating a normalization of intrinsic connectivity patterns in autistic individuals who have less difficulty remembering faces.

## 2. Materials and Methods

### 2.1. Participants

Healthy young adults with a diagnosis of ASD (*n* = 23) and age-, sex-, and IQ-matched healthy typically developing young adults (TD; *n* = 30) were recruited from the community. Exclusion criteria included contraindications to MRI, a history of head trauma, seizure disorders, full-scale IQ < 80, and a current or prior DSM-IV Axis I psychiatric disorder (other than a diagnosis of ASD), including current or a history of substance or alcohol abuse or dependency. Written informed consent approved by the Institutional Review Board at the University of Washington was obtained from all study participants.

ASD diagnoses were confirmed with the Autism Diagnostic Interview-Revised (ADI-R) [33], Autism Diagnostic Observation Schedule (ADOS) [34], and clinical judgment based on all available information and DSM-IV criteria. Intellectual functioning was measured with the Weschler Abbreviated Scale of Intelligence (WASI) [35]. During a semi-structured interview, participants were screened and excluded for current or past DSM-IV-defined psychiatric disorders, or history of a developmental learning disability for TD participants.

Our final sample included 17 individuals with ASD and 22 TD individuals after 14 participants were excluded because of uncorrectable motion or scanner artifacts (4 ASD, 7 TD), full-scale IQ < 80 (2 ASD), and an ADHD diagnosis in a TD participant. The ASD and TD groups did not significantly differ in age, sex, verbal IQ, performance IQ, or full-scale IQ [35]. Specific clinical and demographic data are reported in Table 1.

### 2.2. Face Memory Assessments

The *Faces* subtest of the Wechsler Memory Scale, Third Edition [36] was administered to all participants to assess immediate face memory performance. Participants were asked to remember 24 neutral faces, which were presented sequentially (2 s per picture). The participants were then immediately presented with a set of 48 face photos (consisting of the 24 original faces and 24 novel faces that were randomly shuffled and then presented sequentially). After each picture, the participant was asked if they remembered the face from the previous set, and each correct and incorrect response was recorded. Total scores were converted to scaled scores for analysis.

### 2.3. MRI Data Acquisition

All MRI data were acquired on a 3T Phillips Achieva MR dual Quasar gradient scanner (version 1.5, Philips Medical Systems, Best, The Netherlands) using an 8-channel SENSE head coil.

#### 2.3.1. Anatomical Scan

A T1-weighted anatomical image was acquired in the axial plane for co-registration and anatomical localization using an MPRAGE sequence (magnetization-prepared rapid gradient echo; TR = 7.7 ms; TE = 3.7 ms; flip angle = 8; FOV = 220 mm; matrix 200 × 200; 180 slices; foldover direction RL; REST slab 57.1 mm slice thickness, acquisition voxel size (mm) = 1.00 × 1.00 × 1.00; reconstruction voxel size (mm) 0.86 × 0.86 × 1.00; TFE shots = 144; TFE durations = 1633.0; inversion delay (TI) 823.8 ms).

#### 2.3.2. Functional Localizer Scan

**Figure 1:**
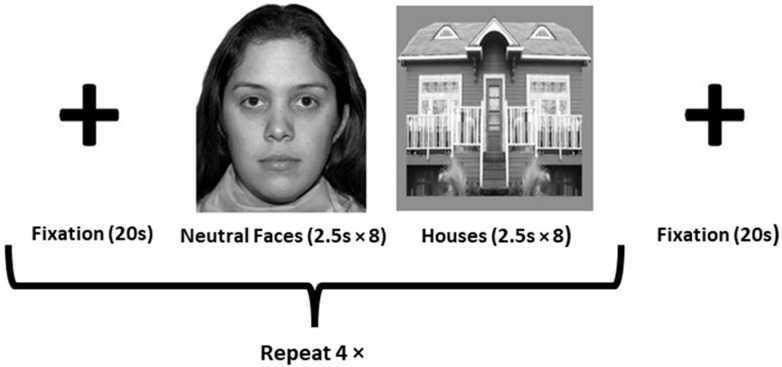
Depiction of face-processing task used to as a functional localizer for the fusiform face area (FFA) and fusiform object area (FOA). The facial stimuli used were obtained, with permission, from the NimStim Set [37].

Whole-brain T2*-weighted images were acquired (TR = 2000 ms; TE = 21 ms; flip angle = 90°; FOV = 240 mm; in-plane resolution = 3.75 × 3.75 mm) to define face- and object-sensitive seed regions. Thirty-two axial slices (slice thickness = 4 mm, 0 mm gap) were acquired per volume (130 volumes total). The functional localizer run involved passively viewing three stimulus types: grayscale neutral faces, houses, and fixation crosses. The stimuli were presented in a block design format in the order depicted in Figure 1.

#### 2.3.3. Resting State Scans

Participants were instructed to “close your eyes, relax, and let your mind wander” prior to scan acquisition. Whole-brain functional MRI volumes were collected using a T2*-weighted single-shot gradient-recalled echo planer imaging (EPI) sequence (FOV = 220 mm, flip angle = 76°, TR = 2000 ms; TE = 21 ms) with a matrix size of 64 × 64 (in-plane resolution = 3.4375 × 3.4375 mm). Each functional volume included 38 contiguous axial slices (slice thickness = 3.5 mm, no gap). Five dummy scans were collected to allow the signal to reach a steady state, followed by 200 volumes (scan duration 6′40″). A B0 field map was collected for distortion correction using a fast field echo sequence (TR = 200 ms; TE1 = 4.6 ms; TE2 = 5.6 ms; flip angle = 30°; FOV = 220 mm, matrix size of 64 × 64, in-plane resolution = 3.44 × 3.44 mm, slice thickness = 3.5 mm, no gap). Scan duration = 53 s. The phase images from the two TE image acquisitions were used to reconstruct the B0 field. The final output included both a magnitude map and a B0 map.

### 2.4. Physiological Monitoring

We monitored and collected physiological data to control for low-frequency cardiac and respiratory contamination and possible aliasing on the fMRI functional connectivity analyses [38,39]. A respiratory belt was placed around the chest, and a pulse oximeter was placed on the index finger of the left hand. Heartbeat and respiration were sampled at 500 Hz and were recorded using LabVIEW™ v 8.6.

### 2.5. fMRI Preprocessing

Both the face localizer and resting-state fMRI data were processed using FSL (FMRIB’s Software Library) 5.0 [37] and AFNI (Analysis of Functional NeuroImages) [38]. Our pipeline includes: (1) motion correction in conjunction with brain extraction and B0 unwarping with FUGUE, (2) physiological correction using 3dRETROICOR (resting state only), (3) filtering out motion parameters and single point motion regressors to control for volumes with 0.5 mm translation from the previous volume on any axis, or absolute translation of 2 mm or 0.5 mm rotation (4) non-brain removal using BET [39], (5) temporal smoothing with a high-pass filter of sigma = 50 s to remove linear drift, and (6) spatial smoothing using a Gaussian kernel of FWHM = 5 mm.

### 2.6. fMRI Processing and Statistical Analysis

**Figure 2:**
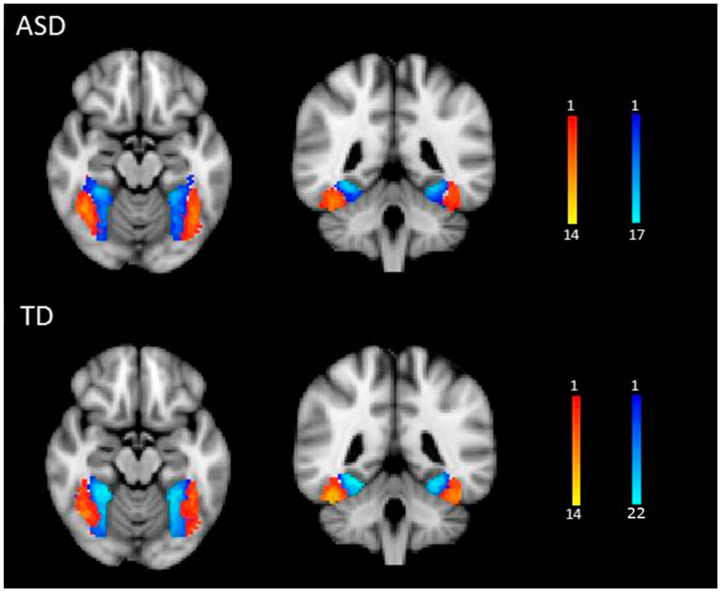
Seed regions in ASD and TD groups. FFA (red-yellow) and FOA masks (blue) show the number of participants who overlapped in the location of the seed regions. Images are presented in radiological convention (L = R).

Four seed regions were identified in each participant from the face localizer scan (Figure 1): the right and left FFA and the right and left fusiform object area (FOA). The FOA was chosen as a control seed region to control for general object processing. A general linear model was specified for the localizer run, with faces and objects as predictor variables. Images showing a fixation cross served as the baseline condition. Data were convolved using a gamma hemodynamic response function. Condition effects were estimated at each voxel within the fusiform gyrus for the contrasts Face > House (FFA) and House > Face (FOA) for each participant using FILM (FMRIB’s Improved Linear Model) [40]. The FFA and FOA seed region masks were created by thresholding the relevant z-stat images at z > 2.3 and binarizing them. For illustrative purposes, we warped each participant’s FFA and FOA masks into standard spaces and created a heat map showing the degree of individual variability in the location of the seed regions (see Figure 2).

Individually specific FFA and FOA masks were applied to each participant’s resting-state scan and the average time series was extracted from these seed regions. Functional connectivity analyses for the right and left hemisphere seed regions were tested separately, with the FFA and FOA time series entered simultaneously in order to statistically control for the intrinsic connectivity associated with general object processing. First-level resting-state analyses of the FFA and FOA seed regions were contingent upon the successful definition of the ROI in the functional localizer scans. One participant with ASD was excluded from further analyses because he did not have significant activation to faces in either the left or the right fusiform gyrus and one participant with ASD was excluded in the left, but not the right, FFA analyses due to a lack of significant activation to faces in the left fusiform gyrus.

Registration for high-resolution structural and standard space images was carried out using FLIRT [41]. FMRI data processing was carried out using FEAT (FMRI Expert Analysis Tool) v 6.0 [40], a part of FSL [37]. Higher-level analysis was carried out using a mixed effects model (FLAME) [42,43]. To investigate group differences in intrinsic connectivity, we tested for group differences in connectivity with the left and right FFA (controlling for FOA).

In addition, we tested correlations with memory performance for each group separately and conducted a test of the group-by-memory performance interaction effect. Z (Gaussianised T/F) statistic images were thresholded using clusters determined by z > 2.3 and a whole-brain corrected cluster significance threshold of *p* < 0.05 [44].

## 3. Results

### 3.1. Face Memory Performance

Significant group differences in immediate face memory performance were found (Table 2), with decreased performance in the ASD group compared to the TD group (ASD mean scaled-score = 7.67 (1.78), range = 4–10; TD mean scaled-score = 10.05 (2.97), range = 6–15, *t*(37) = −2.99, *p* < 0.001). Independent t-tests were conducted to determine if there were significant between-group differences in the types of errors committed in the immediate face recognition task. A significant between-group difference was observed for total hits and misses, *t*(37) = −2.39, *p* = 0.022, but not for the total number of false alarms and correct rejections, *t*(37) = −1.15, *p* = 0.26.

### 3.2. fMRI Results

#### 3.2.1. Motion Parameters

An independent samples t-test was used to test between-group differences in the average root mean square of the absolute motion (movement relative to the fiducial time point) across the entire run. No between-group differences in the root mean square of the motion parameters were present, mean TD = 0.28 (0.26), mean ASD = 0.33 (0.2), *t*(37) = 0.82, *p* = 0.42; range of motion ASD = 0.09–0.89, range of motion TD = 0.10–1.49.

#### 3.2.2. Intrinsic Connectivity Group Difference Analyses

**Figure 3:**
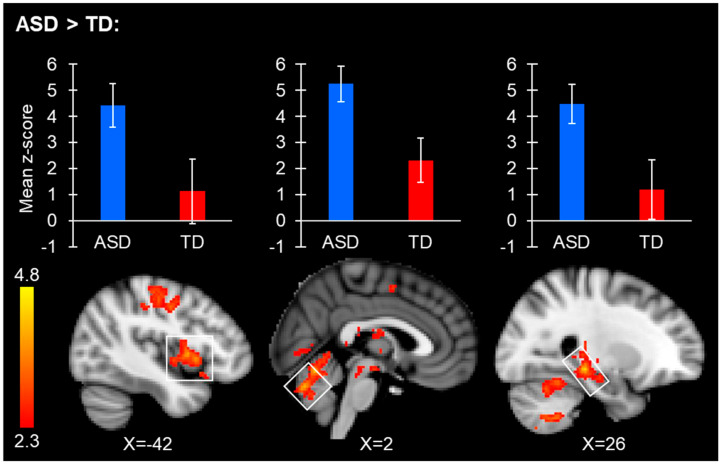
Brain regions with significantly increased right FFA resting-state connectivity in ASD. Bar plots display the average connectivity mean z-score of the clusters located within the white boxes for the ASD (blue) and TD (red) groups separately, and are meant for visualization purposes only. FMRI clusters display peak regions where significant between-group connectivity differences were found. The bar plot and the image showing a group difference in connectivity for the cluster spanning putamen and insular regions can be observed on the left side of the image. The bar plot and connectivity image for the group difference cluster spanning the right cerebellar vermis V/VI are in the middle of the image. Bar plot and connectivity images spanning the hippocampus/thalamus can be observed on the right side of the image.

Both the ASD and TD groups showed extensive connectivity from the right and left FFA area seed regions, with significant connectivity present throughout the entire brain. The ASD group showed increased right FFA connectivity with the hippocampus, amygdala, ventral tegmental area, primary sensory cortex, insular cortex, putamen, cerebellar regions, and thalamus (Table 3/Figure 3). The TD group did not have significantly increased right FFA connectivity with any region. No significant group differences were observed from the left FFA.

#### 3.2.3. Relationship Between Connectivity and Immediate Face Memory Performance

**Figure 4:**
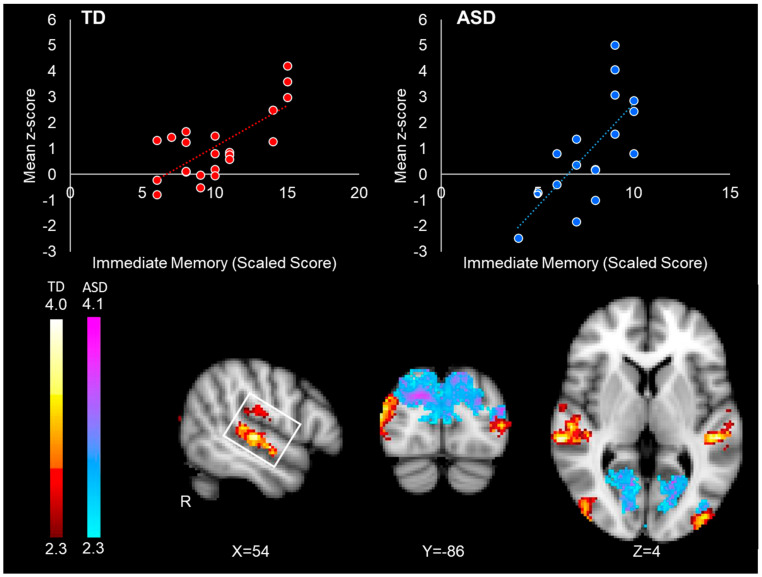
Correlations between right FFA connectivity and immediate face memory performance in the TD group (red-yellow) and the ASD group (blue-purple). The top left red scatterplot illustrates the relationship between FFA connectivity with the right superior temporal gyrus (white box) and face memory scores in the TD participants. The top right blue scatterplot illustrates the relationship between right FFA connectivity with the occipital cortex and face memory performance in the ASD participants. Mean z-score value for each dot was obtained by averaging all the z-score values in the significant connectivity cluster for each specific participant. Scatterplots are for visualization purposes only.

In the ASD group, increased right FFA intrinsic connectivity with the occipital pole, cuneus, precuneus, lingual gyrus, and lateral occipital cortex was associated with better immediate face memory performance (Table 4/Figure 4). In the TD group, higher levels of intrinsic connectivity with the superior and middle temporal gyrus, lateral occipital cortex, and the planum temporale were associated with better immediate face memory performance (Table 4/Figure 4). The relationship between left FFA connectivity and face memory performance was centered in the precuneus in the ASD group and in the lateral occipital cortex in the TD group (see Table 4). No significant inverse correlations between memory performance and FFA connectivity were found in either group.

#### 3.2.4. Group–Performance Interaction

**Figure 5:**
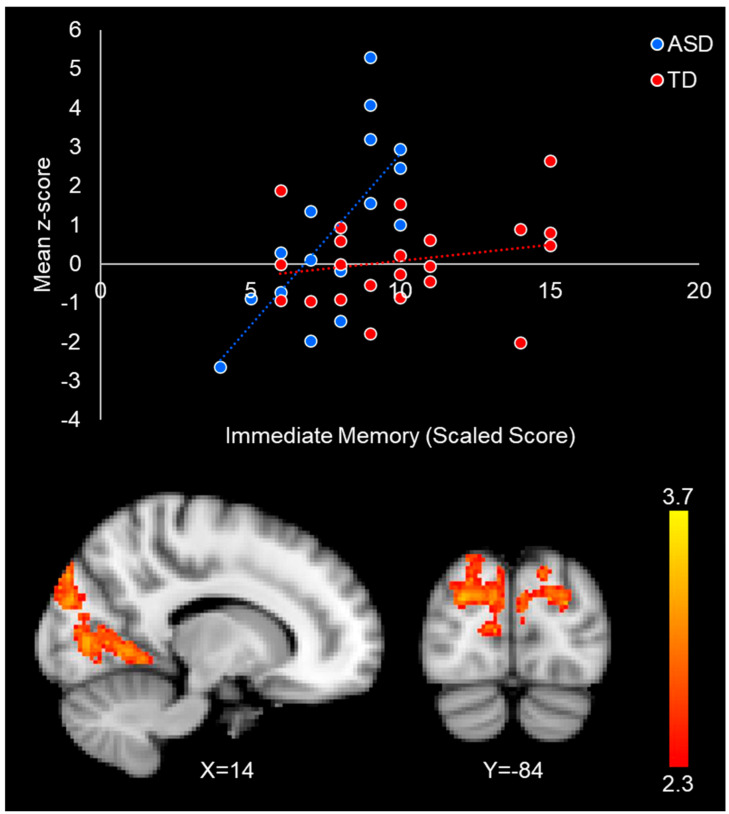
Group by immediate face memory performance interaction in the occipital cortex. The scatterplot illustrates the differential relationship between immediate face memory and the connectivity between the FFA and lateral occipital cortex in ASD (blue) and TD (red) participants. Mean z-score values were obtained by averaging all the z-score values in the significant interaction cluster for each participant. Scatterplots are for visualization purposes only.

We found a significant group–performance interaction effect, such that stronger intrinsic connectivity between the right FFA and occipital lobe (including the cuneus, lateral occipital cortex, lingual gyrus, and precuneus) was correlated to better face memory performance in autistic individuals, but not in the typically developing participants (Table 5/Figure 5). There were no significant interaction effects between immediate face memory performance and group with the left FFA.

## 4. Discussion

The main goal of this study was to investigate the intrinsic connectivity of the neural circuitry supporting face processing and the relationship between intrinsic FFA connectivity and face memory in ASD using a task-free resting-state approach. The neural response to faces is modulated by cognitive factors such as attention, visual imagery, and emotion; as such, our approach allowed us to determine to what extent the connectivity within, between, and outside the core and extended face-processing networks was atypical in autism with potential behavioral confounds removed [45].

The FFA is part of the core face-processing network, along with the inferior occipital gyrus (IOG) and the superior temporal sulcus (STS). In typically developing populations, fixed facial feature identification is processed by the FFA and the IOG [46,47,48]. The superior temporal sulcus, on the other hand, is involved in analyzing gaze direction and speech movements [49,50]. The core face-processing network operates in conjunction with the extended face-processing network, a set of structures that are involved in aspects of socioemotional perception such as visual representation, memory, emotion, and semantics. The core occipitotemporal areas (FFA, STS, and IOG) first perceive and process visual information. Emotion- and reward-processing structures such as the amygdala, the insula, the thalamus, the striatum, the orbitofrontal cortex, the medial frontal gyrus, the inferior frontal gyrus, and the anterior paracingulate link stimulus perception to the socioemotional significance and reaction the stimulus. The hippocampus, parahippocampal area, anterior temporal cortex, precuneus, and posterior cingulate are involved with memory and semantics. The widespread areas of activation during face processing call for analyses that focus on FFA connectivity with both the core and extended face-processing networks and the possible effects of altered connectivity on social dysfunction in ASD.

Connectivity findings in ASD tend to depend on methodological choices such as seed selection, field of view, task regression, and the use of low-pass filtering [51]. In selecting our seed region, we used a reliable functional localizer approach [52] to delineate each participant’s FFA and included the FOA as a control region in our model to control for general object processing. We then performed a group analysis using a whole-brain approach, which allowed us to identify differences in connectivity from the FFA to core and extended face-processing networks, as well as aberrant connectivity from the FFA to areas outside of the core and extended face-processing networks. We hypothesized that individuals with ASD would exhibit decreased intrinsic connectivity between the FFA and the other regions of the core and extended face-processing networks based on research showing FFA underconnectivity during face-processing tasks [18,21]. However, our data showed greater intrinsic connectivity in ASD compared to controls between the right FFA and the insula, amygdala, thalamus, hippocampus, ventral tegmental area, and cerebellum, brain structures that are part of the extended face-processing network [10]. This finding is inconsistent with the literature on FFA connectivity during task performance but is consistent with the literature reporting that patterns of intrinsic overconnectivity may be related to social deficits in ASD [28,53,54] and impaired episodic memory [55]. It is possible that overconnectivity between the FFA and the extended face-processing system may reflect delayed or incomplete functional segregation between the core and extended face-processing networks in ASD [29]. This process of neural specialization progresses with age in typically developing individuals [56], and increased connectivity between the FFA (core) and the extended face-processing network possibly reflects the atypical development of the neural specialization process.

Our work replicated previous studies on impaired face memory performance in autistic individuals [3,57,58,59] and extended previous work by providing evidence that increased connectivity with primary sensory regions that are not part of the face-processing network may contribute to face processing challenges. In the TD group, better face memory performance was observed in individuals with stronger coupling between the right FFA and core face-processing regions: the superior temporal gyrus bilaterally and the temporal occipital fusiform gyrus [10]. Conversely, in the context of impaired face memory in the ASD group as a whole, better face memory performance was observed in autistic individuals with stronger connectivity between the right FFA and the superior and medial regions of the occipital cortex (lateral occipital cortex, cuneus, occipital pole, and lingual gyrus). These regions of the occipital lobe are involved in general visual and object processing and visual feature extraction [60,61], but not face perception, which is localized in the inferior occipital face area [48]. Connectivity with the extended face-processing system was not associated with better face memory performance in either group.

Various factors may limit the implications of our findings. First, our data focus on the functional connectivity of the FFA, a region that plays a specific/limited, albeit critical, role in face processing. Secondly, the generalizability of our results may be limited by our small homogenous ASD sample that comprised primarily males with high cognitive ability. Third, a more in-depth analysis of behavioral measures may be necessary. The measure used to analyze immediate face memory uses only neutral faces to avoid adding a component of emotional cognition to the task. However, this limits our ability to determine whether emotional processing affects face memory performance. Moreover, additional non-face memory tests would benefit our study, in order to discern whether the memory impairments reported in this study are specific to face memory.

To summarize, the current study finds FFA overconnectivity with extended face-processing areas during rest in ASD. Face memory performance was associated with stronger connectivity between the FFA and core face-processing areas in TD participants, whereas autistic individuals with better face memory ability show stronger connectivity to distinct areas involved in general object processing. Taken together, the present findings lend support to previous theories stating that abnormal FFA connectivity may influence face processing and subsequently social function in individuals with ASD. The etiology behind altered connectivity patterns in ASD remains unknown but may be related to developmental abnormalities in the functional specialization of extended face-processing areas and/or to early hyperexpansion of cortical regions during early development in ASD. A longitudinal investigation by Hazlett et al. [62] suggests that individuals with high familial risk for ASD display significant expansion of the bilateral occipital gyrus, the lingual gyrus, and the cuneus, in addition to other cortical areas, between 6 and 12 months of age. Such expansion was found to be highly predictive of an eventual ASD diagnosis. Notably, those regions that show hyperexpansion in the first year of life also show an atypical relationship between connectivity to the FFA and face memory performance in ASD in our current study. Future studies may benefit by studying white matter connections between the FFA and face-processing brain regions as it is possible that differences in structural connectivity are linked to face processing dysfunction in ASD. Studying connectivity from the STS and IOG during rest may also serve to increase our understanding of the neuroanatomic differences in face-processing networks in ASD.

## 5. Conclusions

In conclusion, the present analysis provides evidence for atypical connectivity between face-processing networks in ASD in the absence of potential confounds stemming from face-processing tasks. In contrast to task-based fMRI studies on face processing, we found hyperconnectivity between the FFA and brain regions involved in processing emotion, memory, and visual perception in ASD. Our study also linked FFA connectivity patterns with poor face memory performance in ASD, showing that better face memory performance in ASD is seen in participants who show FFA resting connectivity with occipital areas associated with basic visual perception and general object processing. Coupled with abnormalities in FFA connectivity during face-processing tasks [18,21] and a link between FFA connectivity and social dysfunction [18] and early hyperexpansion [62] in temporoparietal areas, which are linked to poor face memory performance in our current study, our findings point to a possible neurodevelopmental mechanism by which individuals come to exhibit face processing challenges that are prevalent in autistic individuals.

## Figures and Tables

**Table 1:** Demographic and clinical characteristics of included participants.

	ASD	TD	*p*-Value
Group Size	17	22	
Age (SD)	25.1 (4.78)	27.22 (8.19)	=0.35
WASI Full-Scale IQ	112.00 (13.25)	112.57 (12.04)	=0.89
WASI Verbal IQ	109.35 (18.26)	111.67 (11.04)	=0.63
WASI Performance IQ	112.41 (11.01)	110.43 (12.72)	=0.62
ADI-R Social Score	19.41 (5.64)		
ADI-R Communication Score	15.65 (3.66)		
ADOS Severity Score	5.38 (1.75)		
ADOS Social Interaction	5.71 (1.69)		
ADOS Comm. and Language	3.06 (1.48)		

Note. WASI = Wechsler Abbreviated Scale of Intelligence, ADI-R = Autism Diagnostic Interview-Revised. ADOS = Autism Diagnostic Obervation Schedule, Comm = communication.

**Table 2:** Face memory test results.

Immediate Face Memory			
	ASD	TD	
	M(SD)	M(SD)	*p*-Value
Total (Scaled Score)	7.67 (1.78)	10.05 (2.97)	<0.001
Hits	15.71 (3.10)	18.14 (3.20)	0.02
Misses	8.29 (3.10)	5.86 (3.20)	0.02
Correct Rejections	18.47 (2.43)	19.50 (3.00)	0.26
False Alarms	5.53 (2.43)	4.50 (3.00)	0.26

**Table 3:** Group differences in intrinsic FFA connectivity.

Seed	Contrast	Voxels	Peak Region	*p*-Values	z-Max	MNI (mm)			Other Regions
						x	y	Z	
R FFA+	ASD > TD	6596	Left VI	<0.001	4.87	−24	−70	−22	Right Thalamus; Lingual Gyrus; Bilateral Hippocampus; Left Amygdala; Ventral Tegmental Area; Right VIIIa; Right VI
		1207	Precentral Gyrus	<0.001	3.94	−14	−10	58	Postcentral Gyrus; Juxtapositional Lobule Cortex (formerly Supplementary Motor Cortex)
		915	Central Opercular Cortex	<0.001	3.91	−42	6	4	Insular Cortex; Left Putamen; Frontal Orbital Cortex; Precentral Gyrus

Note. R = right, FFA = fusiform face area, MNI = Montreal Neurological Institute. Regions are labeled using the Harvard–Oxford cortical and subcortical atlases. *p*-values are based on whole-brain volume cluster correction for multiple comparisons.

**Table 4:** Correlation between FFA connectivity and immediate face memory performance.

Seed	Group	Voxels	Peak Region	*p*-Values	z-Max	MNI (mm)			Other Regions
						x	y	z	
R FFA+	ASD	6606	Occipital pole	<0.001	4.12	20	−94	28	Lateral Occipital Cortex, superior division; Cuneal Cortex; Intracalcarine Cortex; Lingual Gyrus; Precuneous Cortex
R FFA+	TD	1528	Lateral Occipital Cortex	<0.001	5.02	58	−28	6	Occipital Pole; Supramarginal Gyrus, posterior division; Angular Gyrus
		985	Superior Temporal Gyrus	<0.001	3.95	−34	−68	34	Central Opercular Cortex; Planum Temporale; Middle Temporal Gyrus, posterior division
		844	Lateral Occipital Cortex	<0.001	4.04	56	−22	2	none
		520	Planum Temporale	0.01	3.94	46	−82	12	Superior Temporal Gyrus, posterior division; Central Opercular Cortex
		393	Middle Temporal Gyrus	0.047	3.87	−50	−30	4	Temporal Occipital Fusiform Cortex; Inferior Temporal Gyrus, temporooccipital part; Temporal Fusiform Cortex, posterior division
L FFA+	ASD	538	Precuneus Cortex	0.016	3.79	−14	−62	18	Intracalcarine Cortex; Cuneal Cortex; Supracalcarine Cortex; Lingual Gyrus;
L FFA+	TD	907	Lateral Occipital Cortex	<0.001	3.63	48	−84	14	Temporal Occipital Fusiform Cortex; Occipital Fusiform Gyrus

Note. R = right, L = left, FFA = fusiform face area, MNI = Montreal Neurological Institute. Regions are labeled using the Harvard–Oxford cortical and subcortical atlases. *p*-values are based on whole-brain volume cluster correction for multiple comparisons.

**Table 5:** Interaction between group and face memory performance and FFA connectivity.

Seed	Contrast	Voxels	Peak Region	*p*-Values	z-max	MNI (mm)			Other Regions
						x	y	z	
R FFA+	Group x Face Memory	2583	Cuneal Cortex	<0.001	3.78	−20	−78	30	Lateral Occipital Cortex; Occipital Pole; Intracalcarine Cortex; Lingual Gyrus; Precuneous Cortex

Note. R = right, FFA = fusiform face area, MNI = Montreal Neurological Institute. Regions are labeled using the Harvard–Oxford cortical and subcortical atlases. *p*-values are based on whole-brain volume cluster correction for multiple comparisons.

## Data Availability

The data presented in this study are available on request from the first author. The data are not publicly available due to privacy restrictions.
